# Supplementary material for: Hydrogenotrophic methanogens of the mammalian gut: Functionally similar, thermodynamically different—A modelling approach
Source: PLoS One. 2019 Dec 11;14(12):e0226243. doi: 10.1371/journal.pone.0226243 (PMC6905546; doi:10.1371/journal.pone.0226243)
Supplement: S1 Table — (DOCX) [file pone.0226243.s001.docx]

**Supporting Information**

Hydrogenotrophic methanogens of the mammalian gut: functionally similar, thermodynamically different - A modelling approach

Rafael Muñoz-Tamayo^1*,¶^, Milka Popova^2, ¶^, Maxence Tillier^2^, Diego P. Morgavi ^2^, Jean-Pierre Morel ^3^, Gérard Fonty ^3^, Nicole Morel-Desrosiers^3^

^1^UMR Modélisation Systémique Appliquée aux Ruminants, INRA, AgroParisTech, Université Paris-Saclay, 75005, Paris, France

^2^Institute National de la Recherche Agronomique, UMR1213 Herbivores, Clermont Université, VetAgro Sup, UMR Herbivores, Clermont-Ferrand, France

^3^Université Clermont Auvergne, CNRS, LMGE, F-63000 Clermont-Ferrand, France

S1 Table. Methanogens growth media composition (modified from DSMZ medium 119 <https://www.dsmz.de/microorganisms/medium/pdf/DSMZ_Medium119.pdf> . Growth media was distributed in Balch tubes (6 ml per tube), tubes were sealed and sterilized by autoclaving at 121°C for 20 min. Media preparation and distribution was realized under CO_2_ flushing to assure anoxic conditions. Oxygen traces from commercial gases were scrubbed using a heated cylinder containing reduced copper [1].

| **Composition per 100 ml** | **Amount** |
| --- | --- |
| Clarified rumen fluid ^1^ | 30 ml |
| Dipotassium phosphate (K_2_HPO_4_) 0.06% (w/v) | 5 ml |
| Balch Mineral solution ^3^ | 5 ml |
| Tryptone | 0.2 g |
| Yeast extract | 0.2 g |
| Balch oligo-elements solution ^4^ | 1 ml |
| Balch vitamin solution^5^ | 1 ml |
| Resazurin 0.1%^2^ | 1 ml |
| Ammonium chloride | 0.05 g |
| Sodium acetate | 0.25 g |
| Sodium formate | 0.25 g |
| Sodium carbonate | 0.5g |
| L-cystein HCl^2^ | 0.4g |
| Distilled water | qs 100 ml |

^1^ Rumen fluid, that was the main constituent of the culture medium, was sampled through the rumen cannula from a grazing dairy cow prior to the beginning of the experiment. Sampled rumen contents were strained through a monofilament cloth, the filtrate was then centrifuged at 5 000 g for 15 min. The supernatant was autoclaved and centrifuged again at the same conditions as above. The clarified rumen fluid (decanted supernatant) was stored at -20°C and centrifuged again after thawing prior to media preparation.

^2^ Media was boiled to expel dissolved oxygen, a reducing agent (L-cystein) and a redox indicator (resazurin) were added to keep a low redox potential and indicate the oxidative state of the medium, respectively.

^3^ KH_2_PO_4._2H_2_O (0.6g), (NH_4_)_2_SO_4_ (0.6g), NaCl (1.2g), MgSO_4_.7H_2_O (0.12g), CaCl_2_.2H_2_O (0.12g), distilled water qs 100 ml

^4^ Nitrilotriacetic acid (0.15 g), MgSO_4_.7H_2_O (0.3g), MnSO_4_.2H_2_O (0.05g), NaCl (0.1g), FeSO_4_.7H_2_O (0.01g), CoSO_4_ (0.01g), CaCl_2_.2H_2_O (0.01g), ZnSO_4._2H_2_O (0.01g), CuSO_4_.5H_2_O (0.001g), AlK(SO_4_)_2_ (0.001g), H_3_BO_3_ (0.001g), NaMoO_4_.2H_2_O (0.001g), NiCl.6H_2_O (0.01g), Na_2_SeO_3_ (0.001g), distilled water qs 100 ml

^5^ Biotine (0.2 mg), PABA (0.5 mg), Riboflavine (0.5 mg), Pantothenic acid (0.5 mg), Sodium ascorbate (0.5 mg), Folic acid (0.2 mg), Niacin (0.5 mg), Pyridoxine (0.10 mg), thiamine (0.05 mg), Vitamin B12 0.1mg/ml (0.1 ml), lipoic acid (0.5 mg), Choline chloride (0.5 mg), Inositol (0.5 mg), Nicotinamide (0.5 mg), Pyridoxal (0.5mg), distilled water qs 100 ml

**References**

1. Wolfe RS. Techniques for cultivating methanogens. Methods Enzymol. 2011;494: 1–22.
