# Supplementary material for: Hydrogenotrophic methanogens of the mammalian gut: Functionally similar, thermodynamically different—A modelling approach
Source: PLoS One. 2019 Dec 11;14(12):e0226243. doi: 10.1371/journal.pone.0226243 (PMC6905546; doi:10.1371/journal.pone.0226243)
Supplement: S2 Table — (DOCX) [file pone.0226243.s002.docx]

**Supporting Information**

Hydrogenotrophic methanogens of the mammalian gut: functionally similar, thermodynamically different - A modelling approach

Rafael Muñoz-Tamayo^1*,¶^, Milka Popova^2, ¶^, Maxence Tillier^2^, Diego P. Morgavi ^2^, Jean-Pierre Morel ^3^, Gérard Fonty ^3^, Nicole Morel-Desrosiers^3^

^1^UMR Modélisation Systémique Appliquée aux Ruminants, INRA, AgroParisTech, Université Paris-Saclay, 75005, Paris, France

^2^Institute National de la Recherche Agronomique, UMR1213 Herbivores, Clermont Université, VetAgro Sup, UMR Herbivores, Clermont-Ferrand, France

^3^Université Clermont Auvergne, CNRS, LMGE, F-63000 Clermont-Ferrand, France

S2 Table. Summary of initial OD and pressure measured immediately after primary inoculation. Presented are means (sd) of 31 values per strain.

| Strain | *Methanobrevibacter ruminantium* | *Methanobrevibacter  smithii* | *Methanobacterium formicium* |
| --- | --- | --- | --- |
| Initial OD | 0.054 (0.007) | 0.099 (0.027) | 0.046 (0.01) |
| Initial pressure (mbar) | 2996 (39.956) | 2927 (98.985) | 2910 (48.883) |
|  |  |  |  |
