# Supplementary material for: Hydrogenotrophic methanogens of the mammalian gut: Functionally similar, thermodynamically different—A modelling approach
Source: PLoS One. 2019 Dec 11;14(12):e0226243. doi: 10.1371/journal.pone.0226243 (PMC6905546; doi:10.1371/journal.pone.0226243)
Supplement: S3 Table — (DOCX) [file pone.0226243.s003.docx]

**Supporting Information**

Hydrogenotrophic methanogens of the mammalian gut: functionally similar, thermodynamically different - A modelling approach

Rafael Muñoz-Tamayo^1*,¶^, Milka Popova^2, ¶^, Maxence Tillier^2^, Diego P. Morgavi ^2^, Jean-Pierre Morel ^3^, Gérard Fonty ^3^, Nicole Morel-Desrosiers^3^

^1^UMR Modélisation Systémique Appliquée aux Ruminants, INRA, AgroParisTech, Université Paris-Saclay, 75005, Paris, France

^2^Institute National de la Recherche Agronomique, UMR1213 Herbivores, Clermont Université, VetAgro Sup, UMR Herbivores, Clermont-Ferrand, France

^3^Université Clermont Auvergne, CNRS, LMGE, F-63000 Clermont-Ferrand, France

**S3 Table.** qPCR quantification of 16S rRNA genes

|  | *Methanobrevibacter smithii* | | | | | | |
| --- | --- | --- | --- | --- | --- | --- | --- |
| Time point (hours after inoculation) | 3 | 5 | 6.5 | 24 | 26 | 30 | 72 |
| Microbial pellet (g) * | 0.204 (±0.007) | 0.16 (±0.017) | 0.147 (±0.003) | 0.194 (±0.002) | 0.182 (±0) | 0.176 (±0.012) | 0.202 (±0.012) |
| Extracted DNA ng/g of microbial pellet* | 9765.5 (±3078) | 12695.1 (±838.4) | 13752.8 (±2662.3) | 41257.4 (±25296.9) | 31983.2 (±512.5) | 49386.3 (±3956.8) | 28943.7 (±7300.7) |
| Total number of 16S rRNA gene copies in the microbial pellet | 5.62x10^8^ | 4.34x10^8^ | 2.71x10^8^ | 2.43x10^9^ | 2.68x10^9^ | 2.66x10^9^ | 2.45x10^9^ |
| Total number of cells in the microbial pellet ** | 2.81x10^8^ | 2.17x10^8^ | 1.35x10^8^ | 1.21x10^9^ | 1.34x10^9^ | 1.33x10^9^ | 1.22x10^9^ |
|  | *Methanobrevibacter ruminantium* | | | | | | |
| Time point (hours after inoculation) | 3 | 5 | 6.5 | 24 | 26 | 30 | 72 |
| Microbial pellet (g) * | 0.148 (±0.048) | 0.134 (±0.006) | 0.183 (±0.013) | 0.161 (±0.006) | 0.153 (±0.039) | 0.196 (±0.004) | 0.164 (±0.023) |
| Extracted DNA ng/g of microbial pellet* | 8764.7 (±1345.4) | 10864.3 (±1325.5) | 7546.3 (±1126.5) | 22274.2 (±16) | 19412.8 (±4976.2) | 16184.1 (±578.6) | 23463.5 (±2053.3) |
| Total number of 16S rRNA gene copies in the microbial pellet | 1.52x10^8^ | 1.74x10^8^ | 2.29x10^8^ | 1.82x10^9^ | 9.95x10^8^ | 1.23x10^9^ | 2.06x10^9^ |
| Total number of cells in the microbial pellet ** | 7.62x10^7^ | 8.71x10^7^ | 1.15x10^8^ | 9.10x10^8^ | 4.97x10^8^ | 6.16x10^8^ | 1.03x10^9^ |
|  | *Methanobacterium formicium* | | | | | | |
| Time point (hours after inoculation) | 3 | 5 | 6.5 | 24 | 26 | 30 | 72 |
| Microbial pellet (g) * | 0.13 (±0.017) | 0.167 (±0.017) | 0.086 (±0.003) | 0.15 (±0.064) | 0.167 (±0.044) | 0.195 (±0.016) | 0.153 (±0.052) |
| Extracted DNA ng/g of microbial pellet* | 19517.7 (±3588.2) | 13263.9 (±875) | 21530.9 (±7188.2) | 63810.6 (±28842.7) | 52442.7 (±21309.7) | 38602.9 (±2761.3) | 60968.8 (±28872.4) |
| Total number of 16S rRNA gene copies in the microbial pellet | 2.05x10^8^ | 2.31x10^8^ | 2.60x10^8^ | 1.81x10^9^ | 2.02x10^9^ | 2.42x10^9^ | 3.19x10^9^ |
| Total number of cells in the microbial pellet | 2.05x10^8^ | 2.31x10^8^ | 2.60x10^8^ | 1.81x10^9^ | 2.02x10^9^ | 2.42x10^9^ | 3.19x10^9^ |

* Mean and standar deviation of two observations. ** *M. smithii* and *M. ruminantium* possess two copies of 16S rRNA gene in their genomes.
