# Supplementary material for: Hydrogenotrophic methanogens of the mammalian gut: Functionally similar, thermodynamically different—A modelling approach
Source: PLoS One. 2019 Dec 11;14(12):e0226243. doi: 10.1371/journal.pone.0226243 (PMC6905546; doi:10.1371/journal.pone.0226243)
Supplement: S4 Table — (DOCX) [file pone.0226243.s004.docx]

**Supporting Information**

Hydrogenotrophic methanogens of the mammalian gut: functionally similar, thermodynamically different - A modelling approach

Rafael Muñoz-Tamayo^1*,¶^, Milka Popova^2, ¶^, Maxence Tillier^2^, Diego P. Morgavi ^2^, Jean-Pierre Morel ^3^, Gérard Fonty ^3^, Nicole Morel-Desrosiers^3^

^1^UMR Modélisation Systémique Appliquée aux Ruminants, INRA, AgroParisTech, Université Paris-Saclay, 75005, Paris, France

^2^Institute National de la Recherche Agronomique, UMR1213 Herbivores, Clermont Université, VetAgro Sup, UMR Herbivores, Clermont-Ferrand, France

^3^Université Clermont Auvergne, CNRS, LMGE, F-63000 Clermont-Ferrand, France

## S4. Calculation of thermodynamic properties of the methanogenesis

In our model, the methanogenesis is represented macroscopically by one catabolic reaction (R1) for methane production and one anabolic reaction (R2) for microbial formation. We assumed that ammonia is the only nitrogen source for microbial formation. The molecular formula of microbial biomass was assumed to be C_5_H_7_O_2_N [1].

R_1_: 4 H_2_ + CO_2_ 🡪 CH_4_ + 2 H_2_O

R_2_: 10 H_2_ + 5 CO_2_ + NH_3_ 🡪 C_5_H_7_O_2_N + 8 H_2_O

The thermodynamic properties associated to the methanogenesis result from the contribution of both catabolic and anabolic reactions.

**Thermodynamic properties of formation used for the calculations**

**S4 Table.** Standard enthalpies (Δ*H*_f_°) and Gibbs energies (Δ*G*_f_°) of formation at 25°C of compounds involved in hydrogenotrophic methanogenesis. Values were extracted from Wagman et al. [2], with the exception of the microbial biomass that was calculated from values for *Methanosarcina barkeri* reported by Liu et al. [3]

| Compound (phase) | Δ*H*_f_° (kJ/mol) | Δ*G*_f_° (kJ/mol) |
| --- | --- | --- |
| H_2_0 (l) | -285.830 | -237.129 |
| H_2_ (g) | 0 | 0 |
| CO_2_ (g) | -393.509 | -394.359 |
| CH_4_ (g) | -74.81 | -50.72 |
| NH_3_ (aq) | -80.29 | -26.50 |
| C_5_H_7_O_2_N | -511.50^*^ | -349.50^*^ |

^*^ These values are five times the values reported by Liu et al. [3] since the biomass formula we used has five carbon molecules, while the biomass formula used by Liu et al. has one carbon.

**Enthalpies**

The heat produced during methanogenesis results from the contribution of both catabolic and anabolic reactions. So, first, we calculated the standard enthalpies of the catabolic and anabolic reactions using the standard enthalpies of formation given in S4 Table for the different compounds involved in methanogenesis.

The standard enthalpy of the catabolic reaction $\Delta H_{r,c}^{^{\circ}}$was calculated as follows

$$\Delta H_{r,c}^{^{\circ}}=\Delta H_{f,\mathrm{CH}_{4}}^{^{\circ}}+2\cdot\Delta H_{f,H_{2}O}^{^{\circ}}-\left( 4\cdot\Delta H_{f,H_{2}}^{^{\circ}}+\Delta H_{f,\mathrm{CO}_{2}}^{^{\circ}} \right)=-252.96\frac{\mathrm{kJ}}{\mathrm{mol}}$$

A similar equation was used for the calculation of the standard enthalpy of the anabolic reaction $\Delta H_{r,a}^{^{\circ}}$

$$\Delta H_{r,a}^{^{\circ}}=\Delta H_{f,C_{5}H_{7}O_{2}N}^{^{\circ}}+8\cdot\Delta H_{f,H_{2}O}^{^{\circ}}-\left( 10\cdot\Delta H_{f,H_{2}}^{^{\circ}}+5\cdot\Delta H_{f,\mathrm{CO}_{2}}^{^{\circ}}+\Delta H_{f,NH_{3}}^{^{\circ}} \right)=-750.31\frac{\mathrm{kJ}}{\mathrm{mol}}$$

These results are at 25°C since this is the temperature of the standard enthalpies of formation reported in S3 Table. A correction could be made to get results at 39°C but the heat capacities reported by Wagman et al. [2] show that the temperature correction can be neglected. Similarly, in the interest of simplicity, we assumed that the effect of pressure is negligible. Next, we considered the fact that the heat of a given reaction can be calculated at any state along the reaction pathway *via* the determination of the reaction coordinate or degree of advancement $\varepsilon$ [4]. Under our assumptions, the heat produced or consumed by a particular reaction during a given interval can be calculated as follows

$$Q= \int_{\varepsilon_{0}}^{\varepsilon_{t_{f}}} \Delta H_{r}^{^{\circ}}d\varepsilon$$

For our two reactions, at the instant $t$ we have

$$\varepsilon_{c}\left( t \right)= \frac{n_{H_{2}}\left( t \right)-f\cdot n_{H_{2,0}}}{-4}$$

$$\varepsilon_{a}\left( t \right)= \frac{n_{H_{2}}\left( t \right)-\left( 1-f \right)\cdot n_{H_{2,0}}}{-10}$$

where $n_{H_{2}}\left( t \right)$is the number of moles of hydrogen at the instant $t$, $n_{H_{2,0}}$ is the initial number of moles of hydrogen, and $f$ is the fraction of H_2_ used for the catabolic reaction. Our calorimetric experiments started with $n_{H_{2,0}}=8.83\cdot{10}^{-5}$mol in all cases. At the final time $t_{f}$, all the hydrogen was consumed, so that $n_{H_{2}}\left( t_{f} \right)=0$. For *M. smithii* and *M. ruminantium*, the microbial yield factor is $Y$=0.006 (6) which implies that $f=$0.94. Accordingly, $\varepsilon_{c}=2.075\cdot{10}^{-5}$ mol and $\varepsilon_{a}=5.30\cdot{10}^{-7}$ mol. It thus follows that the overall heat produced during the methanogenesis process ($Q_{m}$) can be calculated using the following equation

$$Q_{m}=Q_{c}+Q_{a}= \varepsilon_{c}(t_{f})\cdot\Delta H_{r,c}^{^{\circ}}+\varepsilon_{a}(t_{f})\cdot\Delta H_{r,a}^{^{\circ}}$$

where $Q_{c},Q_{a}$are the heat produced during catabolism and anabolism respectively. The previous equation can also be written as

$$Q_{m}=n_{H_{2,0}} \left[ \frac{\left( 1-10Y \right)}{4} . \Delta H_{r,c}^{^{\circ}}+Y . \Delta H_{r,a}^{^{\circ}} \right]$$

Under the experimental conditions of our study, this yields

$$Q_{m}=Q_{c}+ Q_{a} =\left( -5.25 \right)+ \left( -0.40 \right)= -5.65 J$$

This result shows that the anabolic reaction contributes to only 7% of the metabolic heat.

Since the substrate was totally consumed, the enthalpy of the methanogenesis process per mole (or C-mol) of biomass formed, $\Delta H_{m}$, can be calculated as follows

$$\Delta H_{m}= \frac{Q_{m}}{n_{\mathrm{biomass}}}= \frac{Q_{m}}{Y . n_{H_{2,0}}} = \frac{\left( 1-10Y \right)}{4 . Y} . \Delta H_{r,c}^{^{\circ}}+\Delta H_{r,a}^{^{\circ}}$$

which yields

$$\Delta H_{m}= -10658\frac{\mathrm{kJ}}{\mathrm{mol}}= -2132\frac{\mathrm{kJ}}{C-mol}$$

For *M. formicium*, $Y$=0.007 (7). Applying the same procedure, we obtained $Q_{m}= -5.66 J$ and

$$\Delta H_{m}=-1830\frac{\mathrm{kJ}}{C-mol}$$

The anabolic reaction contributes to 8% of the metabolic heat.

**Gibbs energies and entropies**

Following a procedure analogous to the one used above for the enthalpies, the standard Gibbs energies of the catabolic ($\Delta G_{r,c}^{^{\circ}}$) and anabolic ($\Delta G_{r,a}^{^{\circ}}$) reactions were calculated using the standard Gibbs energies of formation listed in S3 Table.

$$\Delta G_{r,c}^{^{\circ}}= \Delta G_{f,\mathrm{CH}_{4}}^{^{\circ}}+2 . \Delta G_{f,H_{2}O}^{^{\circ}}-\left( 4 . \Delta G_{f,H_{2}}^{^{\circ}}+\Delta G_{f,CO_{2}}^{^{\circ}} \right)= -130.62 \frac{\mathrm{kJ}}{\mathrm{mol}}$$

$$\Delta G_{r,a}^{^{\circ}}= \Delta G_{f,C_{5}H_{2}O_{7}N}^{^{\circ}}+8 . \Delta G_{f,H_{2}O}^{^{\circ}}-\left( 10 . \Delta G_{f,H_{2}}^{^{\circ}}+5 . \Delta G_{f,CO_{2}}^{^{\circ}}+ \Delta G_{f,\mathrm{NH}_{3}}^{^{\circ}} \right)= -248.24 \frac{\mathrm{kJ}}{\mathrm{mol}}$$

The free energy of the methanogenesis process per mole (or C-mol) of biomass formed, $\Delta G_{m}$, can then obtained from the following equation

$$\Delta G_{m}= \frac{\left( 1-10Y \right)}{4 . Y} . \Delta G_{r,c}^{^{\circ}}+\Delta G_{r,a}^{^{\circ}}$$

which yields for *M. smithii* and *M. ruminantium*

$$\Delta G_{m}= -5364 \frac{\mathrm{kJ}}{\mathrm{mol}}= -1073 \frac{\mathrm{kJ}}{C-mol}$$

Knowing that

$$\Delta G_{m}= \Delta H_{m}-T. \Delta S_{m}$$

it follows that the entropic contribution to the methanogenesis process is equal to

$$T.\Delta S_{m}= \Delta H_{m}- \Delta G_{m}= -5294 \frac{\mathrm{kJ}}{\mathrm{mol}}= -1059 \frac{\mathrm{kJ}}{C-mol}$$

which gives, at 39°C, the following value for the entropy of the methanogenesis process per mole (or C-mol) of biomass formed

$$\Delta S_{m}= \frac{(\Delta H_{m}- \Delta G_{m})}{(273.15+39)}= -16.96 \frac{\mathrm{kJ}}{K\mathrm{mol}}= -3.40 \frac{\mathrm{kJ}}{K C-mol}$$

The same procedure applied to *M. formicium* yields $\Delta G_{m}=-917 \frac{\mathrm{kJ}}{C-mol}, \Delta S_{m}=-2.92 \frac{\mathrm{kJ}}{C-mol}$

**References**

1. Batstone DJ, Keller J, Angelidaki I, Kalyuzhnyi S V, Pavlostathis SG, Rozzi A, et al. Anaerobic Digestion Model No.1 (ADM1). IWA Task Group for Mathematical Modelling of Anaerobic Digestion Processes. IWA Publishing, London; 2002.

2. Wagman DD, Evans WH, Parker VB, Schumm RH, Halow I, Bailey SM, et al. The Nbs Tables of Chemical Thermodynamic Properties - Selected Values for Inorganic and C-1 and C-2 Organic-Substances in Si Units. J Phys Chem Ref Data. 1982;11: S2.

3. Liu JS, Marison IW, von Stockar U. Microbial growth by a net heat up-take: A calorimetric and thermodynamic study on acetotrophic methanogenesis by Methanosarcina barkeri. Biotechnol Bioeng. 2001;75: 170–180. doi:Doi 10.1002/Bit.1176

4. Prigogine I, Defay R. Traité de thermodynamique, conformément aux méthodes de Gibbs et De Donder. Éditions Desoer. Liège; 1950.
