# Supplementary material for: Hydrogenotrophic methanogens of the mammalian gut: Functionally similar, thermodynamically different—A modelling approach
Source: PLoS One. 2019 Dec 11;14(12):e0226243. doi: 10.1371/journal.pone.0226243 (PMC6905546; doi:10.1371/journal.pone.0226243)
Supplement: Attachment — Submitted filename: 20191121_Response to Reviewers_final.docx [file pone.0226243.s006.docx]

Dear Dr. James E. Wells Academic Editor PlOS ONE,

We have decided to submit a revised version of the article. Below, we addressed the comments of reviewer.

We hope that you will find the revised version suitable for publication in PLOS ONE.

Sincerely

On behalf of the authors

Rafael Muñoz-Tamayo

**Reviewer #2**:

*I appreciate the rewrite of the introduction by the authors, which definitely made the paper improve in clarity. However, I feel the authors can still make the very final step regarding the added value of the paper to the existing literature. I suggest that the authors add a last piece of information to the discussion section to discuss why the present model development would contribute to increased understanding of the immune system of humans and the prediction of greenhouse gases from the rumen. Or how suggested further model development dealing with nonlinear behaviour related to spatial and temporal variation in mammalian gut systems contributes to this? Do we need to be able to model coexistence to answer these questions?*

**Response:** characterising the dynamics of rumen methanogens (and other rumen microbes) is essential to the enhanced understanding of the microbial community functioning and thus holobiont’s phenotype. In particular, the efficiency of methane mitigation strategies are strongly dependent of dynamic properties such as the rumen ecosystem resilience and also by the functional redundancy of microbes (Weimer, 2015). This functional redundancy is related to the aspects discussed in our article. Our modelling work aimed to characterise methanogen dynamics. We prefer in the paper to avoid being speculative about the impact of our model on the prediction of *in vivo* systems and focus on our actual outcomes avoiding the risk of overselling our findings. On-going and future work will aim at expanding the model to the gut ecosystem, but we are aware that we have a long way.

In regard to the relevance of incorporating nonlinear behaviour related to spatial and temporal variation in mammalian ecosystems, these aspects have been already discussed in our previous developments (Muñoz-Tamayo *et al.*, 2010) and by other authors (Widder *et al.*, 2016). Spatio-temporal mechanisms are determining for the functioning and colonization of the human gut (Labarthe *et al.*, 2019). An experimental work indicated the importance of temporal dynamics to enhance the understanding on rumen function (Huws *et al.*, 2016).

The level of detail of the model is an open question for the modeller and depends of the question the model is intended to help to answer. For example, as demonstrated in our modelling works, we do not need information of the rumen microbiota to predict accurately enteric methane production (Muñoz-Tamayo *et al.*, 2019). However, microbial information is needed into models to inform on manipulation strategies and improving understanding of rumen microbial function as discussed in our recent review (Huws *et al.*, 2018). For the human gut, analysing competition and syntrophy between gut microbes allows to identify assembling rules of the microbial community. “Notably, elucidating the assembly rules of the microbiome goes beyond gaining a better understanding of basic ecological processes and has profound clinical implications.”(Levy and Borenstein, 2013)

These interesting questions should be addressed integrating new models and data from those used in our work.

*I apologise for unclarity from my side regarding the reported CCC and R^2 in Table 3 that seemed unrealistically high. What I meant is that it appears to me that the model parameters were fitted to the data that was available. Using those fitted parameters, predicted values were obtained from model simulations and compared with observed values. This comparison resulted in CCC and R^2 values. My point is that the same data should not be used for model parameter fitting and model evaluation to ensure that model predictions are evaluated independently. If a model is not evaluated independently, observed vs. predicted plots could serve as valid diagnostic plots, but CCC and R^2 values are trivial. Independent data is needed for a solid model evaluation. The authors may split the data that is available if they are able to do so.*

**Response:** we recognize the importance of model validation (falsification). However, model validation is not an exclusive criterion for model evaluation. The lack of the model validation step does not preclude a rigour analysis of model evaluation. The implementation of a model validation requires a large data set. In our study, the number of sampling times for each microbe is limited. Accordingly, splitting the data for calibration and validation is not a good strategy. Reducing the number of data for model calibration will reduce the informative content of the process dynamics and thus will have a detrimental effect in the accuracy of the parameter estimates. Model validation is desired when data is available under other experimental conditions (e.g. operation under continuous mode), which is not the case in our study. We disagree with the analysis the reviewer performs with respect to the statistic indicators for model evaluation. The values of R2 and CCC calculated under the calibration context are not trivial since a model with structural problems will lead to unsatisfactory R2 and CCC. The high values that we obtained for R2 and CCC demonstrated that the model is well structured and that captures the dynamic of the methanogenesis. Our satisfactory calibration outcomes are the result of an adequate model construction that includes the theoretical identifiability property of the model as discussed in the article and also from the fact that we addressed practical identifiability issues. The sampling times were actually determined using an optimal experiment design (OED) strategy to facilitate accurate estimation of the parameters. This OED strategy was based using estimates from preliminary data and implementing an optimization problem that maximizes the determinant of the Fisher Information Matrix (Muñoz-Tamayo *et al.*, 2014). This information is not detailed in the article to avoid the reader to get lost in mathematical technicalities. It should be said that our calibration strategy follows a validation-like principle since the maximum specific growth rate constants were obtained from the calorimetric experiments data and injected further into the dynamic model challenged with the data from the growth kinetics experiments. This procedure strengthens the quality of our model.

In lines 410-413, we recognized the limitation of our work with respect to model validation.

*I wonder why the x-axes of Figures 3 and 4 run over 75 and 100 h, respectively, instead of 75 h for both Figures.*

**Response:** Figure 4 was modified to get the axes homogenous

References

Huws, S.A., Creevey, C.J., Oyama, L.B., Mizrahi, I., Denman, S.E., Popova, M., et al. (2018) Addressing global ruminant agricultural challenges through understanding the rumen microbiome: past, present, and future. *Front Microbiol* **9**: 2161.

Huws, S.A., Edwards, J.E., Creevey, C.J., Stevens, P.R., Lin, W., Girdwood, S.E., et al. (2016) Temporal dynamics of the metabolically active rumen bacteria colonizing fresh perennial ryegrass. *FEMS Microbiol Ecol* **92**:.

Labarthe, S., Polizzi, B., Phan, T., Goudon, T., Ribot, M., and Laroche, B. (2019) A mathematical model to investigate the key drivers of the biogeography of the colon microbiota. *J Theor Biol* **462**: 552–581.

Levy, R. and Borenstein, E. (2013) Metabolic modeling of species interaction in the human microbiome elucidates community-level assembly rules. *Proceeding Natl Acad Sci United States Am* **110**: 12804–12809.

Muñoz-Tamayo, R., Laroche, B., Walter, E., Doré, J., and Leclerc, M. (2010) Mathematical modelling of carbohydrate degradation by human colonic microbiota. *J Theor Biol* **266**: 189–201.

Muñoz-Tamayo, R., Martinon, P., Bougaran, G., Mairet, F., and Bernard, O. (2014) Getting the most out of it: Optimal experiments for parameter estimation of microalgae growth models. *J Process Control* **24**:.

Muñoz-Tamayo, R., Ramírez Agudelo, J.F., Dewhurst, R.J., Miller, G., Vernon, T., and Kettle, H. (2019) A parsimonious software sensor for estimating the individual dynamic pattern of methane emissions from cattle. *Animal* **13**: 1180–1187.

Weimer, P.J. (2015) Redundancy, resilience, and host specificity of the ruminal microbiota: implications for engineering improved ruminal fermentations. *Front Microbiol* **6**: 296.

Widder, S., Allen, R.J., Pfeiffer, T., Curtis, T.P., Wiuf, C., Sloan, W.T., et al. (2016) Challenges in microbial ecology: Building predictive understanding of community function and dynamics. *ISME J* **10**: 2557–2568.
